# Sodium and Potassium

**Published:** 1914-10-15

**Authors:** R. Ottolengui


### SODIUM AND POTASSIUM.

BY R. OTTOLENGUI, D.D.S.

Sodium and Potassium Compound was introduced to the dental profession at the World's Columbian Dental Congress, and in value is worth all the money spent on the congress. It was presented by Dr. Emil Schreier, of Vienna, and the best preparation is that put up under his supervision, and imported. Another preparation put up in this country is in small phials the object of which was to meet the complaint that the agent rapidly disintegrates by oxidation. Hence the smaller the package the less the loss. As a matter of fact the reverse is true, as it is almost impossible to preserve the contents of the small phials once they are opened, so that the final expense is greater than with Shreier's package. This is shaped like a short test tube, the sodium and potassium appearing like an amalgam, covered at the top by about a quarter of an inch of paraffin. In order to reach it, an instrument is used to puncture a hole in the paraffin, thus gaining access to the sodium and potassium compound, but immediately after the treatment of a tooth the hole in the paraffin should be closed by pressing into it a warm burnisher. This prevents the oxygen of the air from reaching it, and as it has a tremendous affinity for oxygen, if this precaution is not taken it will rapidly disintegrate by oxidation.

Sodium and Potassium is not primarily intended for use in canals in which living pulps are present. Nevertheless, in cases where pressure anesthesia has not been entirely satisfactory, or where the removal of the pulp has not been certainly accomplished, the compound may be used to good purpose. It is, of course, most undesirable to leave a terminal filament of the pulp at the apex, since by the following day when all effects of the anesthetic will have passed off, this filament will be found very much alive and exceedingly difficult to anesthetize. Where doubt exists therefore, it has been my practice after controlling the hemorrhage, and before the anesthetic effects have disappeared to use the sodium and potassium compound for extirpating the remains of the pulp, just as one would proceed in the presence of dead tissue.

The method of using sodium and potassium is as follows: Select the finest of the Young Aseptic broaches, and having made a hole through the paraffin, push the broach down into the compound. It will come forth evenly charged with the amalgam-like mass, and must be used immediately, as the compound oxidizes rapidly when exposed to the air. Therefore the rubber dam should be in place, the cavity cleared of all debris and ready access to the opening of the canals must have been procured by freely cutting away the occlusal surface. Let us suppose that the opening to a canal has been found but that a fine clean broach has failed to penetrate more than a quarter of the length of the canal. We desire, with the aid of the compound, to clear out this canal to the apex. The fine broach freshly charged with the compound, is forced into the canal as far as it will go, using an up and down stroke. A rotary movement may also be exerted, but only with the greatest caution. Have the following axiom constantly in mind when working in root canals. *A broach will never break if moved only forward and back in a canal, but may and often does break when rotated.* The reason is simple. If the end jams it can best be dislodged by a straight backward withdrawal. It can often be broken by rotating it. Therefore avoid rotation of a broach as mush as possible.

There is one other thing needed besides the sodium and potassium in reaching the end of a canal. This fine broach naturally carries but a very small dose of the compound to the part of the canal which is filled with partly calcified material. Therefore time is required. The story will be like this. Charge the broach with the compound and pump the instrument gently back and forward a few times. No progress! Cleanse the broach, and recharge it. No progress! Repeat the process. No progress! Repeat the process. No progress! Repeat the process. No progress! Here some impatient men abandon the treatment, and wander around dental meetings declaring: "There is nothing in that sodium and potassium treatment. I have tried it." Other men proceed as follows: Recharge the broach, pump back and forth. "Ah That went a little deeper!" Repeat. "Ah! A little deeper still!" After which the progress is usually satisfactorily rapid. In many cases the calcification of the pulp has built a bridge across the canal which prevents ingress, and it takes some time to break down this mass chemically by the action of the sodium and potassium, but having passed this point the balance of the canal may be fairly easy. And it is exactly this class of unfilled root, that gives trouble, because the dentist by stopping at the bridge, as it were, leaves imprisoned in the canal beyond, a considerable mass of material which is most attractive pabulum for bacterial organisms.

Sometimes, however, the entire canal is filled up with calcific material and there is no such satisfactory result as to find the broach suddenly passing the bridge and plunging forward into comparatively clear space. It is these cases of almost complete calcification that test a man's patience as well as his ability to convince his patient that the service will be worth the time and money expended upon it. Yet both may be bone.

But it is not alone in the presence of calcific material that sodium and potassium is valuable. It is a magnificent agent for sterilizing infected canals, and even may be relied upon to sterilize the tubuli. Its power as a germicidal agent was well proven in a series of tests carried on a few years ago, with the assistance of a competent bacteriologist. The operator was supplied with sterilized glass tubes, into which broaches to be tested were placed by first breaking off one end of a tube and then after introducing the broach resealing the tube by melting the end in an alcohol flame. Some of these broaches were bare broaches used for exploring infected canals. Others were broaches which had been charged with sodium and potassium and then used for sterilizing the canals. Some of these latter were even dipped directly into pus. In all cases cultures were procurable from the dry broaches used, but in no single instance could a culture be obtained from any of the broaches which had been charged with the sodium and potassium.

One should certainly be cautious in young teeth, or any teeth having large foramina. Yet after twenty years' experience with the compound I do not recall a single case of irritation or trouble of any sort. It must be remembered that it is to be used only on the finest of broaches, and that it oxidizes so rapidly that it is frequently difficult to get a good dose of it far up into a canal. Usually, therefore, one need have no anxiety on that score.

It does not cleanse the canal at all. Where calcific material is present it breaks down their solidity so that the particles may be picked out. And where only soft tissue is present by chemical combination it forms a mass almost resembling green soap. After treating a canal which may have appeared clean prior to treatment, it will be a revelation to many to see the debris that may be removed. This after treatment includes syringing with peroxide of hydrogen into which is mixed mercury bi-chloride (1 to 500), after which I prefer to go further and cleanse the canal with whisps of cotton wound on the finest of the Young broaches and dipped in the same peroxide solution. When these bits of cotton are withdrawn they should be removed from the broach onto a nakpin, when they will appear as a green stripe. A new whisp is introduced and the process continued so long as these bits of cotton show any color, after which the syringe should be again used, followed by clean sterile cones of bibulous paper for removing the moisture.—"Around the Table" in *Items of Interest.*

